# Multidimensional analysis of health in Mexico: implementation of fuzzy sets

**DOI:** 10.1186/s12889-021-10988-2

**Published:** 2021-05-19

**Authors:** Lucio Flores-Payan, Diana Mercedes Hernández-Corona, Tonatiuh González-Heredia

**Affiliations:** 1 Public Policy Department, Administrative Economic Sciences University Campus, University of Guadalajara, Zapopan, Jalisco Mexico; 2 Multidisciplinary Health Research Center, Biomedical Science Department, Tonalá University Campus, University of Guadalajara, Tonalá, Jalisco Mexico

**Keywords:** Diabetes mellitus, High blood pressure, Excess weight, Depressive symptoms, Fuzzy sets

## Abstract

**Background:**

The national health and nutrition survey allows to know the state of health of the Mexican population, it provides data for the analysis of different factors and / or indicators of health, diseases and nutritional conditions, such as chronic degenerative diseases and depressive symptoms, which, in turn, if both occur simultaneously, they will have a negative impact on health. This article studies the four factors involved in the overall health of the population in Mexico: excess weight, diabetes, high blood pressure, and depressive symptoms, which are used to conduct a multidimensional characterization and analysis.

**Methods:**

Two methodological resources are applied, a descriptive statistical characterization and the construction of a multidimensional health index with the use of fuzzy sets, through the National Health and Nutrition Survey (ENSANUT 2018–19 - *for its acronym in Spanish*) in Mexico.

**Results:**

The results reveal a growing percentage of individuals who experience detriments to their health, that is, the factors being studied have had a negative impact and tend to follow international projections. The construction of a multidimensional index enables the interaction between the factors being studied, thus allowing for an adequate modeling for the identification of health in Mexico.

**Conclusion:**

This study aims to elucidate the current state of health throughout the population in Mexico by using the most current data provided by the autonomous public body of statistics and geography to build a multidimensional panorama using four elementary public health indicators (diabetes, obesity, high blood pressure, and depressive symptoms).

## Background

The data from the 2018–19 National Health and Nutrition Survey [1], the most important survey at the national level that allows insight into the state of health of the Mexican population at different stages of life, was recently published. This survey provides data for the analysis of different factors and / or indicators of health, diseases and nutritional conditions, among which diabetes, excess weight, high blood pressure and depressive symptoms are the most prevalent [2].

Mexico is the country with the highest prevalence of obesity worldwide, as well as diabetes and high blood pressure [3–5], therefore, measuring this data in Mexico is valid, and different analytical proposals exists for the understanding, measurement and quantification of the factors that promote the deterioration of the health of individuals.

It is important to recognize that depressive symptomatology is not well studied, nevertheless, it has been shown to have a negative impact upon health, particularly when it occurs simultaneously with chronic degenerative diseases such as high blood pressure [6]. Furthermore, it has even been reported that depressive symptoms and high blood pressure are associated with increased morbidity and mortality [7].

Therefore, integrating the analysis of diabetes, excess weight, high blood pressure and depressive symptoms in a multidimensional health index, and observing the prevalence in each federal entity in Mexico, will allow us to know how the population is and its geographical distribution, that is why the objective of this study was to carry out the statistical-descriptive characterization of health factors (diabetes, excess weight, high blood pressure and depressive symptoms), and the construction of a multidimensional health index with the implementation of fuzzy sets, using the information provided within the National Health and Nutrition Survey (ENSANUT – *for its acronym in Spanish*) 2018–19 [1].

## Construction and content

Two methodological resources are applied: a) the descriptive cross-sectional study and b) correlational study, which includes four factors involved in the state of health of Mexicans such as: diabetes, excess weight, high blood pressure and depressive symptoms.

Two methodological resources which are used were both developed around the four factors being considered and complement each other to characterize the overall health status of the population in Mexico.

### Descriptive cross-sectional study

The first methodological resource, a descriptive cross-sectional study, allows for the socio-demographic analysis of these factors through the use of descriptive statistics, with frequencies and percentages for qualitative variables and mean and standard deviation for quantitative variables. It is described by sex and degree of excess weight. The distribution according to the degree of obesity, and its relationship with diabetes, high blood pressure and depressive symptoms in men and women are also described. In addition, the description by socioeconomic level is integrated into quartiles, with 1 being the lowest socioeconomic level and 4 the highest socioeconomic level. The academic level is reported as: none, basic education, upper secondary education, higher education and postgraduate, and is analyzed with each of the four factors being studied: diabetes, high blood pressure, depressive symptoms and excess weight.

Finally, the prevalence of each study factor is shown by state, in such a way that it allows for the visualization of how diabetes, excess weight, high blood pressure and depressive symptoms are distributed at a national level. The SPSS®^□^ software version 23.0 was used for the statistical analysis. Likewise, the prevalence is calculated per 100 individuals with a confidence interval of 95% (CI 95%).

### Correlational study

The second methodological resource, is based around the construction of a multidimensional health index (MHI), with the use of fuzzy sets, with the aim of integrating the four factors being studied and quantifying the distribution of overall health of the Mexican population at the national level.

Given the significant use of fuzzy sets for the creation of the IMS in this study, the usefulness of fuzzy modeling and its application in fuzzy sets is detailed prior to the description of the construction of the IMS along with a brief review of the literature with applications of fuzzy sets in the health area.

### Configuration of the fuzzy modeling

In itself, fuzzy modeling is a series of techniques that make it possible to differentiate variables into rigid and fuzzy, allowing for the creation of multidimensional models without sacrificing the robustness of the results [8]. Rigid variables are those which can be easily segmented: black or white, one peso or one million, health or disease, while fuzzy variables present problems for their categorization; Someone who measures one point eight meters is considered tall, but is someone who only measures one point seven-nine meters not considered so?, or in terms of grade 3 obesity who, according to the WHO, should have a BMI of > 40 kg/m2, how would we then consider someone who is at 39.99 kg/m2? Should this person really be completely excluded from the group of people with grade 3 obesity?

Fuzzy sets allow us to assign a degree of membership between 0 and 1 to a certain set, in the above-mentioned example, the individual with a BMI of 39.99 kg > > would have a degree of membership to the set of individuals with obesity grade 2, however they would also have a degree of belonging to the set of individuals with grade 3 obesity. In traditional logic, this individual would be excluded from the set of individuals with grade 3 obesity, but not with the use of fuzzy sets. This enables a more comprehensive analysis, and allows for focalization by using the data obtained via fuzzy modeling.

Another important factor about the use of fuzzy sets is the ability to disaggregate the variables that make up a model. That is, although the use of fuzzy sets allows for the calculation of multidimensional indices, it also allows for particularity in the analysis of their variables since, by identifying the causal conditions and not only the relationship between them, the particular effects of each variable can be estimated using the output data [8].

Fuzzy modeling is configured using three main elements: 1) Fuzzy Inference System (FIS), 2) Fuzzy sets, 3) Operating rules. The FIS is the representation of knowledge and data and their integration through fuzzy sets. It is built from the non-linear relationship between one or more input variables and an output variable, going through a process of transformation and data recovery.

The fuzzy sets that make up the FIS are a collection of objects, where each one corresponds to a membership function which assigns the degrees of membership to each set. The range of the membership function can be a set of non-negative real numbers, conventionally the membership function is defined between 0 and 1 as X μA → [1,0].

Finally, certain operating rules are required for the correct functioning of the FIS. These are reference parameters which the model will use to perform the calculations. For example, suppose that we incorporate two aspects associated with health and their respective labels: excess weight (normal weight, obesity) and high blood pressure (Yes, No), as well as three linguistic labels to assess the combination of both variables in terms of something that would represent health (good, fair, deteriorated). In this way it would be possible to encode a person with normal weight and without high blood pressure as having a “good” level of health.

### Application of fuzzy sets

Fuzzy sets have primarily been used in engineering, however they have also increasingly been applied to biomedical development and health analysis. Some studies, such as those presented by authors Saltkjel, Holm, Dahl and Halvorsen, which explore the relationship between crises and public health in countries within the European Union, use a model built with the use of fuzzy sets to analyze the changes in the health of the populations these European Union nations [9]. Likewise, the study conducted by Kien, Grillich, Nussbaumer and Schoberberger, analyzes the effect of implementing health improving strategies upong the health of the population using a comparative analysis based on fuzzy sets [10]. Furthermore, Dmour, Sagahyroon and Mli, propose a model of fuzzy sets to analyze causality between the deterioration of the health of a population and associated health problems, in which they conclude that modeling through the use of fuzzy sets makes it possible to focus on health modifiers that are not routinely identified [11].

Having stated the above, in the sense of elucidating the importance of the use of fuzzy sets in this particular study and in the analysis of health conditions in general, the construction of the multidimensional health index will be present, for which it was use a fuzzy inference system (FIS), based on the theory of fuzzy logic and its mathematical materialization of fuzzy sets [8].

**Fig. 1:**
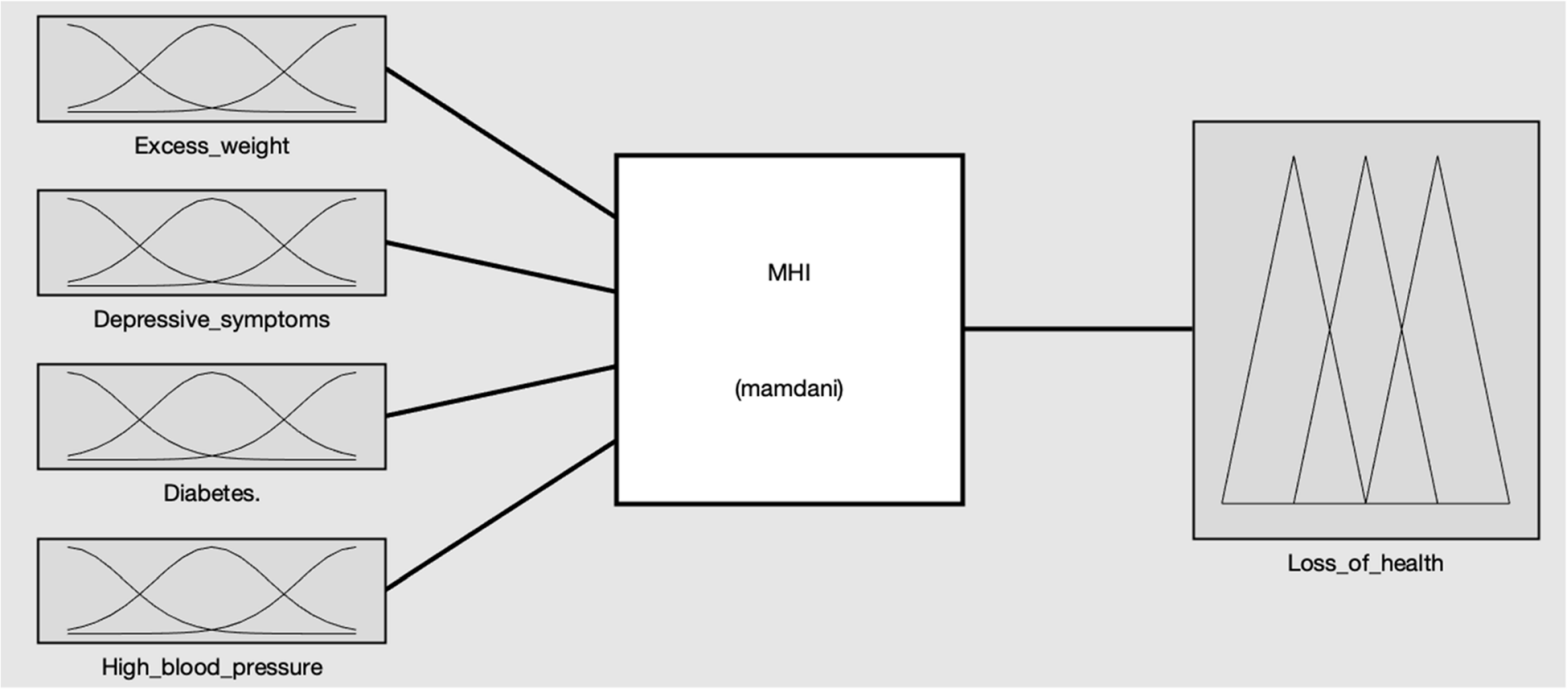
Fuzzy System for calculating the Multidimensional Health Index. Source: created by the authors. MHI: Multidimensional Health Index

**Fig. 2:**
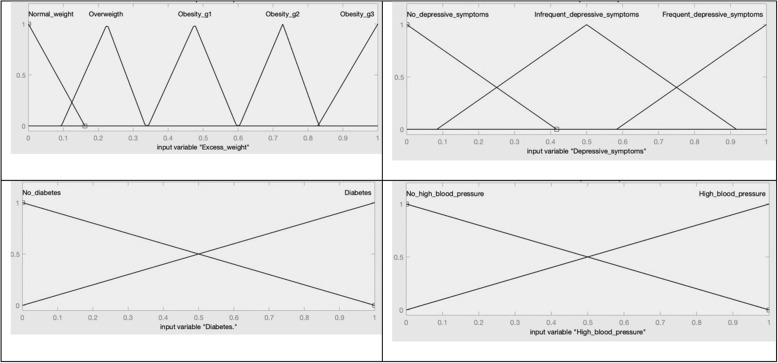
Fuzzy sets for the input variables. Source: created by the authors

**Fig. 3:**
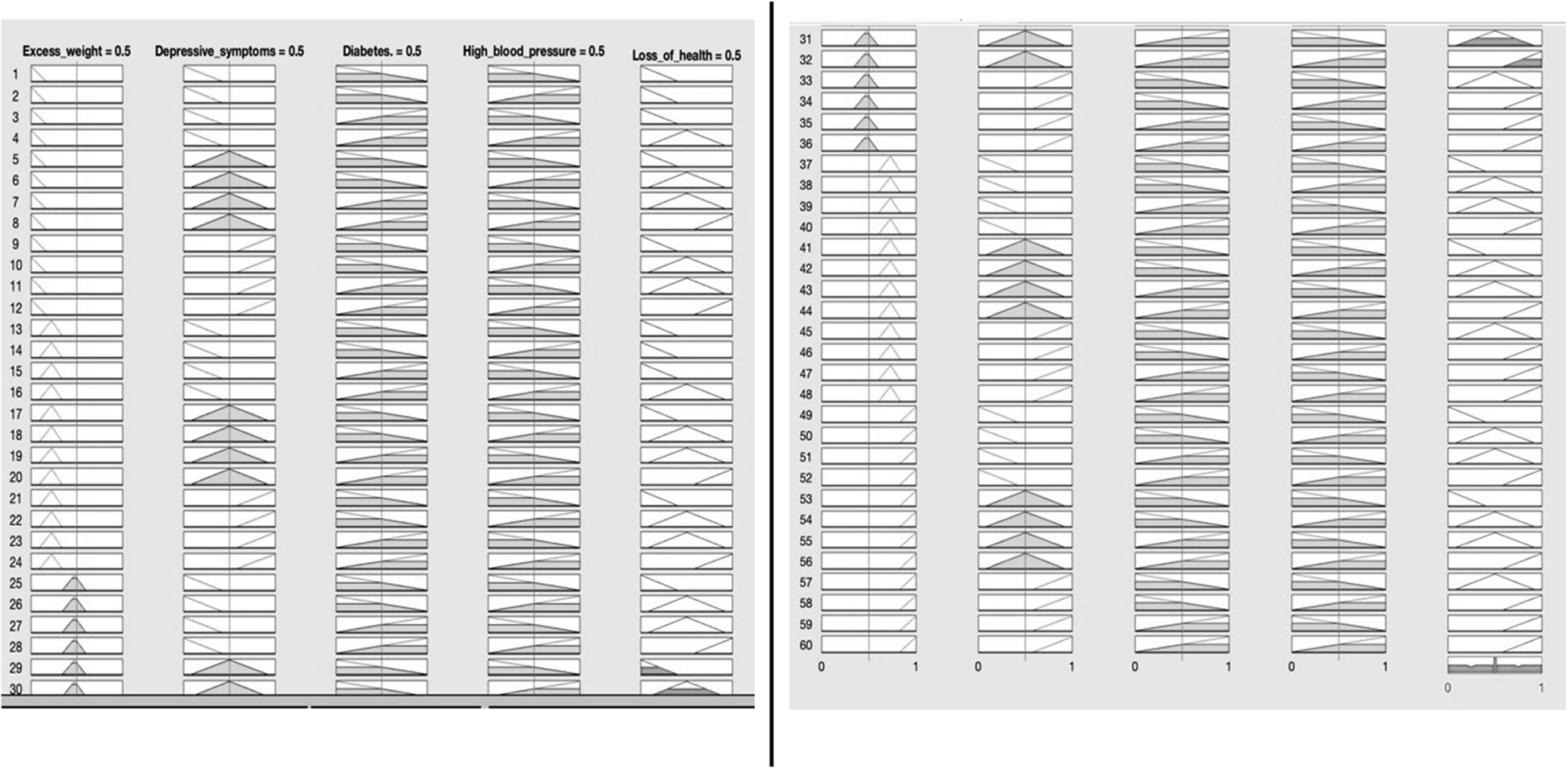
Rules of operation of the fuzzy inference system. Source: created by the authors

The construction of the index was configured using the four factors being studied: diabetes, excess weight, high blood pressure and depressive symptoms; as input variables, and the state of health and / or loss of health, represented by the MHI, were established as quantifying and output variables (See Fig. 1). Likewise, the fuzzy sets of each input variable and the output variable were defined in such a way as to allow each dataset to be positioned on a continuum and quantified by the degree of belonging to the same fuzzy set (see Fig. 2). The FIS was operationalized using the set of 60 operational rules (see Fig. 3), which were validated through substantive knowledge, which consists of the integration of different theoretical, conceptual, empirical and assessment aspects by experts [8]. Through these operating rules, the possible combinations of the factors being analyzed are declared from the measurement of their magnitudes, allowing for the creation of a measuring rule and / or quantifying variable [12]. The MHI was built on a scale of 0–100 where the value of 100 expresses the least favorable state and / or loss of health of each individual of the total being studied. The fuzzy system data was processed using the MATLAB®^□^ Software, its toolbox application was used for the creation of the fuzzy sets.

### Data source

The data source for the statistical-descriptive characterization of the health factors, the comparative analysis and for the construction of the multidimensional health index was the National Health and Nutrition Survey [1] (ENSANUT) 2018–19.This survey is carried out at the national level throughout Mexico, it is comprised of a health and a nutrition component; the sample size is 50,000 homes for the health component and 32,000 for the nutrition component, which together represent 125.5 million inhabitants (using a probabilistic, stratified, and cluster approach), and integrates demographic, socioeconomic, health and nutritional data. The analysis was carried out with 13,140 records of the total sample of the ENSANUT 2018–19, with further expansion to 61′873,020, the former being records with complete data for all the variables used, the rest of the records were not used in either of the two methodological resources.

Regarding the four factors being analyzed, the paragraphs below describe how they were taken from the ENSANUT 2018–19 for the consideration of prevalence.

1. **Diabetes:** in the section for adults, the following question was taken into account, “Has a doctor told you that you have diabetes (or high blood sugar)?” with three answer options 1) yes, 2) yes, during pregnancy (women only, called gestational diabetes), 3) no. Response 1 was considered for the determination of prevalence while 2 and 3 were discarded.
2. **Excess weight:** in the section for adults, the overweight and obesity section, the following question was taken into account, “Has a doctor / dietician / nutritionist ever told you that you are currently or have had obesity?” with 2 possible options: 1) yes, 2) no, answer 1 was taken into account. To complement the nutrition component of the survey, in the anthropometry section, data is provided regarding the average weight and height with the formula for body mass index (BMI): kg / m^2^ and was classified according to the World Health Organization (WHO) as follows: normal weight from 18 to 24.9 kg / m^2^, overweight from 25 to 29.9 kg / m^2^, obesity grade 1 from 30 to 34.9 kg / m^2^, obesity grade 2 35 to 39.9 kg / m^2^, obesity grade 3 > 40 kg / m^2^.
3. **High blood pressure:** in the section for adults, the following question was taken into account, “Has a doctor told you that you have high blood pressure?” with 2 answer options: 1) yes, 2) no. Answer 1 was taken into account.
4. **Depressive symptoms:** in the section for adults within the ENSANUT 2018–19, the following question was considered: “During the last week, did you feel as if you could not rid yourself of sadness?” for which there were 4 possible answers: 1) rarely or never (less than a day), 2) few times or sometimes (1–2 days), 3) a considerable number of times (3–4 days), 4) all of the time or most of the time (5–7 days). Option 1 was considered for the determination of prevalence of no depressive symptoms, and responses 2 to 4, with depressive symptoms.

One of the main limitations in the use of the ENSANUT microdata was the summarized form in which the four study factors are presented in the populational data. Therefore, one of the possible lines of improvement in this health survey would be the use of a new instrument which allows for the construction of more abundant information regarding the health factors being studied.

## Results

### Statistical-descriptive characterization of the factors being studied

Several trends of the factors being studied are distinguished with the intention of presenting a characterization of the determinants of the health status of Mexicans: diabetes, excess weight, high blood pressure and depressive symptoms in relation to sociodemographic elements.

Of the total sample of the ENSANUT 2018–19, diabetes affects 11% of which 9.3% correspond to men and 11.7% to women; In terms of excess weight, about 24% of respondents confirmed having obesity, with 19% correspond to men and 27% to women, however, in accordance with data regarding weight and height of each individual surveyed, 83.2% were found to have excess weight (i.e. overweight, obesity grade 1, 2 and 3). This distribution is shown in Table 1. Other determinants of health, such as high blood pressure, were reported in 19.2% of those being surveyed, with 15.7% being men and 22.0% women. Finally, depressive symptoms were reported in 11.9% of those being surveyed, with 6.7% in men, and 14.6% in women.

**Table 1:** Distribution of excess weight and grades of obesity by sex

Sex	n	Expansion	Overweight		Obesity 1		Obesity 2		Obesity 3	
		×100	%	95% CI	%	95% CI	%	95% CI	%	95% CI
Men	5601	261,079.1	40.1	(38.82,41.38)	27.97	(26.78,29.14)	8.66	(7.92,9.40)	5.18	(4.6,5.76)
Women	7539	357,651.1	34.8	(33.76,35.91)	29.55	(28.52,30.58)	12.79	(12.03,13.54)	6.95	(6.38,7.52)

Table 1, makes it evident that a large percentage of the population can be classified as overweight, given that approximately 70% of the study population falls within the overweight and grade 1 obesity categories.

Table 2 classifies the relationship between excess weight and the three factors (depressive symptoms, diabetes and high blood pressure) by se. It is important to note that depressive symptoms were more prevalent among overweight individuals, and not among those with diabetes and high blood pressure, which were generally categorized as having grade 1 obesity, particularly in the case of men. Women with grade 1 obesity, on the other hand, had the highest prevalence for all factors, that is, depressive symptoms, diabetes and high blood pressure.

**Table 2:** Relationship between the factors: depressive symptoms, diabetes and high blood pressure with the degree of excess weight

	Excess Weight	n	Expansion	Depressive Symptoms n (%)	Diabetes n (%)	High Blood Pressure n(%)
Men	Over -weight	2246	10,479,825	63 (38.2)	121(33)	201 (29.3)
	Obesity 1	1566	7,405,540	41 (24.8)	123 (33.5)	242 (35.2)
	Obesity 2	485	2,098,860	10 (6.1)	48 (13.1)	111 (16.2)
	Obesity 3	290	1,296,897	11 (6.7)	21 (5.7)	70 (10.2)
Women	Over -weight	2626	12,258,330	139 (28.5)	184 (28.6)	319 (26.2)
	Obesity 1	2228	10,446,861	159 (32.6)	220 (34.2)	416 (34.1)
	Obesity 2	964	4,474,253	80 (16.4)	107 (16.6)	225 (18.5)
	Obesity 3	524	2,342,832	41 (8.4)	85 (13.2)	173 (14.2)

Table 3, shows the prevalence of the four factors being studied divided by quartile of declared income. Both excess weight and depressive symptoms are more prevalent in people with higher socioeconomic income, found in the 4th quartile, while high blood pressure and diabetes are more prevalent among individuals of lower socioeconomic levels, with the highest being those in the 1st quartile.

**Table 3:** Population distribution in relation to level of income and the four factors being studied

	Excess Weight				High Blood Pressure			
			95% confidence interval of the difference				95% confidence interval of the difference	
Income quartiles	Average	Mean standard error	Inferior	Superior	Average	Mean standard error	Inferior	Superior
1st quartile	0.2163	[0.00557]	0.2054	0.2272	0.3319	[0.01369]	0.3051	0.3588
2nd quartile	0.2237	[0.00588]	0.2122	0.2353	0.2609	[0.0131]	0.2352	0.2866
3rd quartile	0.2303	[0.00582]	0.2189	0.2417	0.2295	[0.01211]	0.2057	0.2533
4th quartile	0.297	[0.00677]	0.2837	0.3103	0.2326	[0.01149]	0.2101	0.2552
	Diabetes				Depressive Symptoms			
			95% confidence interval of the difference				95% confidence interval of the difference	
Income quartiles	Average	Mean standard error	Inferior	Superior	Average	Mean standard error	Inferior	Superior
1st quartile	0.1173	[0.00435]	0.1088	0.1258	0.6092	[0.0066]	0.5962	0.6221
2nd quartile	0.0715	[0.00364]	0.0644	0.0787	0.6701	[0.00664]	0.657	0.6831
3rd quartile	0.0607	[0.0033]	0.0542	0.0672	0.7302	[0.00613]	0.7181	0.7422
4th quartile	0.0636	[0.00361]	0.0565	0.0707	0.8127	[0.00578]	0.8013	0.824

**Fig. 4:**
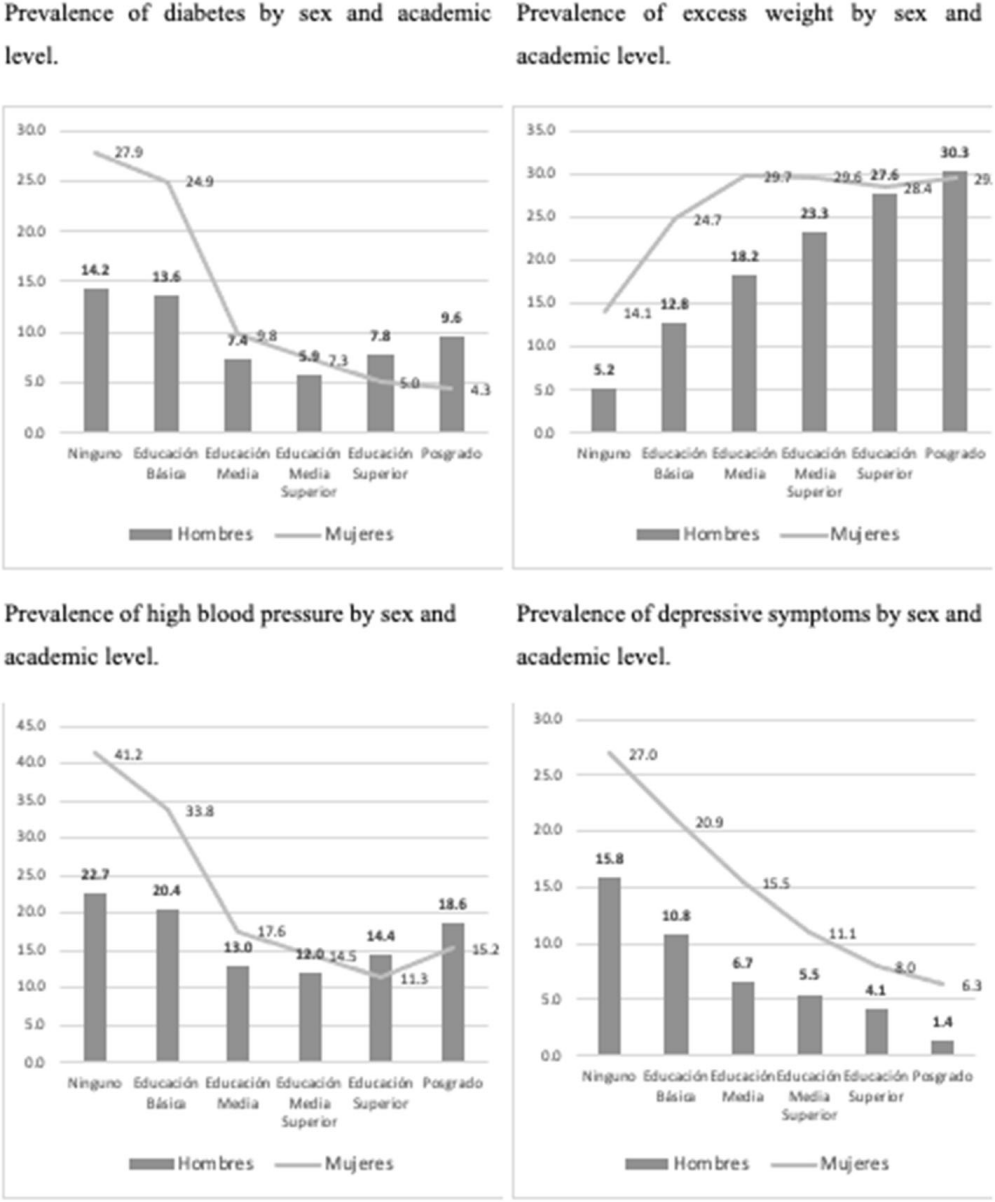
Prevalence of the four factors analyzed. Source: created by the authors data from ENSANUT 2018–19

When observing the relationship between the four factors being studied and academic level reported by the surveyed Mexican population, a decreasing trend becomes evident for three of the factors (i.e. diabetes, high blood pressure and depressive symptoms), Fig. 4, from lower to higher level of studies. In other words, individuals with lower academic levels tend to have higher rates of diabetes, high blood pressure and depressive symptoms. On the other hand, excess weight has the opposite relationship, with higher rates among both men and women with postgraduate degrees. It shows that diabetes doesn’t decrease as academic level increases among men as it does with women, in whom a notable decrease occurs among those with postgraduate degrees.

### The following paragraphs demonstrate the distribution of the prevalence of these four factors at the national level by state

**Fig. 5:**
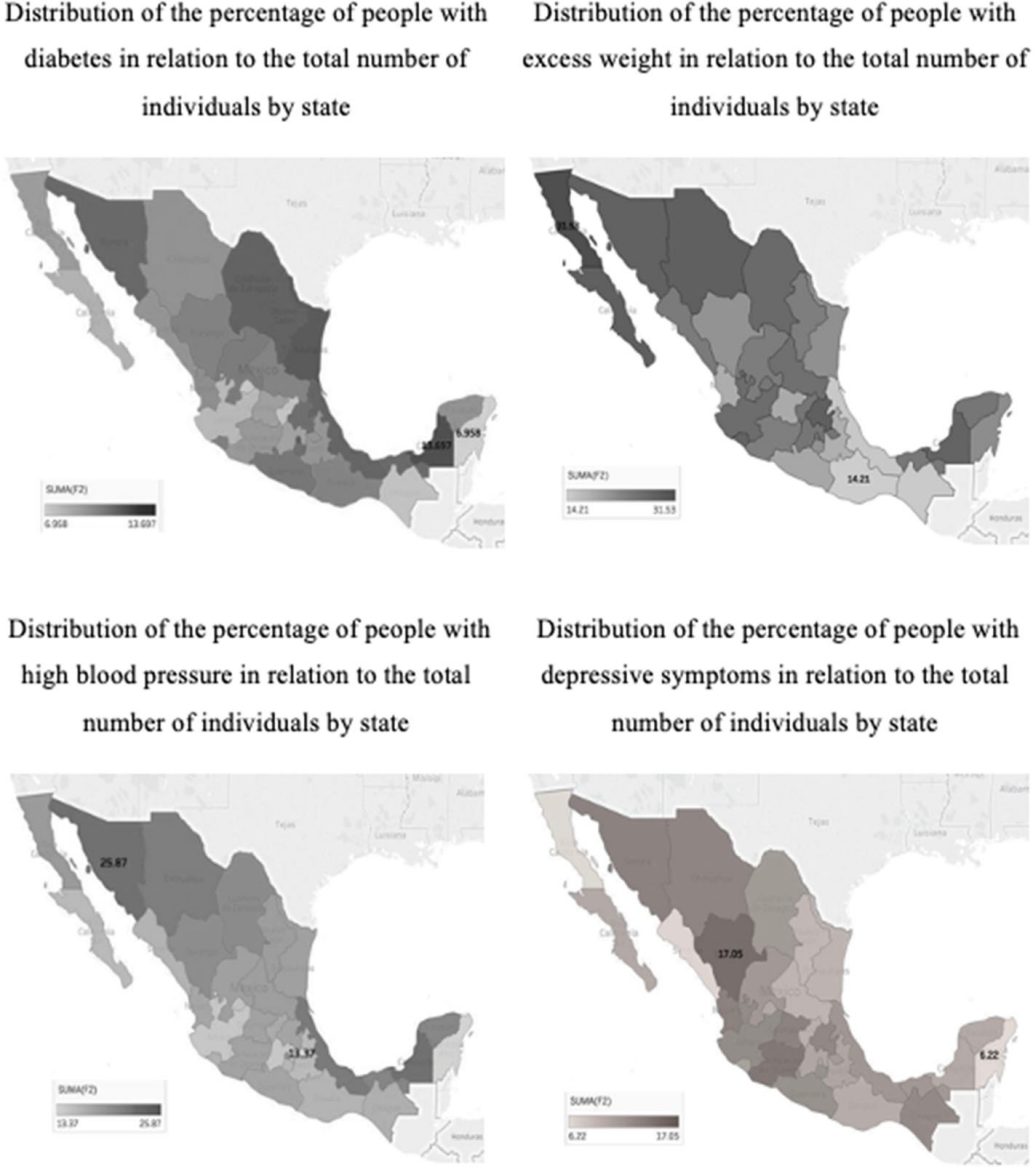
Distribution of the percentage of people in relation with four factors analyzed and Multidimensional Health Index average by state. Source: created by the authors with data from ENSANUT 2018

The distribution of the four factors by the state is shown in Fig. 5. States around the Gulf of Mexico (i.e. Durango, Tamaulipas, Veracruz and Campeche) show the highest prevalence for diabetes, ranging from 7.0 to 13.7% of the studied population in Yucatán and Campeche respectively, with the national average being 11.0%. Shows that obesity is more prevalent among the norther states, with Baja California having the highest number of individuals with this condition with 31.5%, while Oaxaca has the lowest number with just 14.2%, and the overall national average is 24%. It is important to note that this distribution was made based on what the individuals responded in the ENSANUT 2018–19 and not with their weight and height data.

Furthermore, demonstrates that the nationwide prevalence of high blood pressure is generally low, however, states such as Sonora, Chihuahua, Veracruz, Campeche and Quintana Roo, have the highest concentration of individuals with this condition, with Sonora particularly being the state with the highest prevalence, reported at 25.9%, while Tlaxcala has the lowest rates with just 13.4%. The national average for high blood pressure was reported as 19.2%.

In addition, shows that the distribution of depressive symptoms is somewhat homogeneous nationwide, with Durango being the state with the highest percentage (17.0%), Yucatán being the lowest (6.2%) and the national average being 11.9%.

#### The following describes the construction of a multidimensional health index (MHI) with the use of fuzzy sets which will show how health is distributed around Mexico

The construction and calculation of the MHI enabled the quantification of a variable which allows for the integration of the 4 factors under study in order to demonstrate the loss of health. Due to the nature of its construction using fuzzy sets, this index is flexible and allows for the integration of more factors to increase the accuracy of the characterization and measurement of the loss of health of individuals with a decreased loss in explanatory level [13].

Table 4, shows the distribution of the MHI and its mean value for different classificatory variables, with groups of individuals without studies having a higher degree on the MHI. Likewise, the northern region of the country also shows a higher degree of this index compared to the other three under study, loss of health is also evident to a greater degree among individuals with factors such as alcohol and tobacco consumption.

**Table 4:** Construction of the Multidimensional Health Index (MHI)

	Average MHI	95% confidence interval of the difference	
		Inferior	Superior
**Sex**			
Man	20.3365	(19.9594	20.7136)
Woman	23.8980	(23.4945	24.3015)
**Level of Education**			
No education	29.0121	(26.9548	31.0693)
Basic level	25.9039	(25.2304	26.5774)
Middle education	22.2467	(21.7709	22.7224)
Higher secondary education	20.2803	(19.7627	20.7980)
Higher education	18.9692	(18.4015	19.5369)
Postgraduate	19.8958	(17.7479	22.0438)
**Access to Medical Service**			
With Access	20.4114	(19.8960	20.9268)
No Access	22.0444	(21.6203	22.4686)
**Access to private health insurance**			
With Access	20.5188	(19.1652	21.8725)
No Access	21.5909	(21.2439	21.9379)
**Population Type**			
Urban	22.5224	(22.1707	22.8741)
Rural	22.0987	(21.6208	22.5766)
**Regions**			
North	23.1174	(22.4768	23.7580)
Central	21.4628	(21.0244	21.9012)
Mexico City	22.5513	(20.9278	24.1747)
South	22.8346	(22.3660	23.3032)
**Consumes alcoholic beverages**			
Yes	23.6620	(23.0863	24.2377)
No	20.7997	(20.3955	21.2039)
**Smoking**			
Every day	22.6178	(21.5263	23.7093)
Some days	22.6687	(22.3517	22.9856)
Does not smoke	19.9181	(19.1780	20.6583)
**Age**			
20_30	17.1278	(16.8092	17.4464)
31_40	19.9937	(19.5450	20.4424)
41_50	24.2371	(23.6404	24.8338)
Greater than 50	31.3044	(30.4337	32.1751)

*MHI* Multidimensional Health Index.

**Fig. 6:**
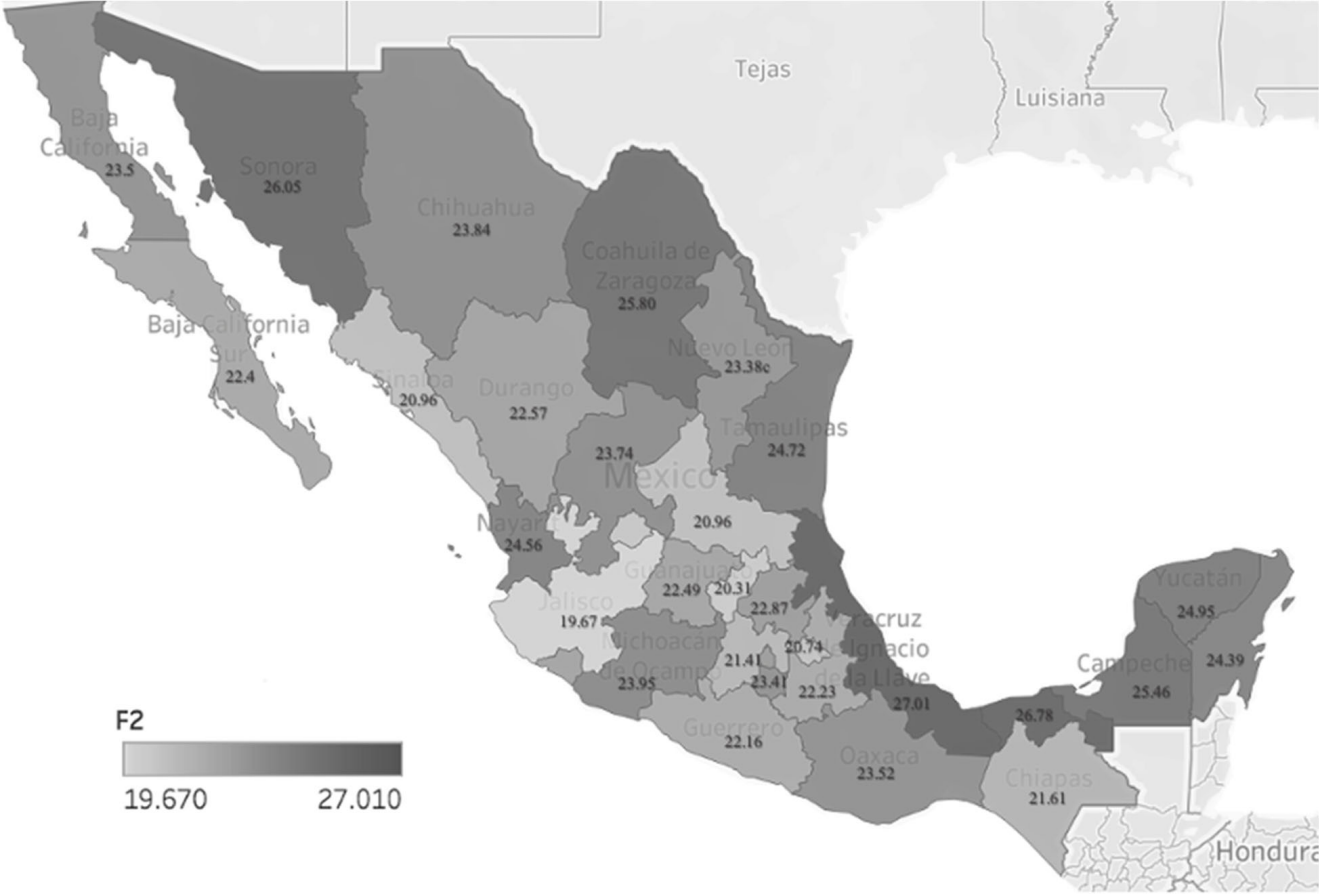
Distribution of the MHI average, by state. Source: created by the authors, with data from ENSANUT 2018–19

Figure 6, shows the distribution of the mean value of the MHI by state, where a significant gap can be observed in the loss of health of the inhabitants of certain northern states and the Gulf of Mexico, which show a higher prevalence on the MHI. The state with the highest prevalence was Veracruz with 27.0 on the MHI, and the state with the lowest MHI was Jalisco with 19.7, with a national average of 22.4 on the MHI, remembering that 0 represents the optimal health condition and 100 represents a loss of health.

## Discussion

The integration of methodological strategies makes it possible to show elements of great interest, such as the clear trend of increasing prevalence in the pathologies being analyzed, caused by four of the main public health problems: diabetes, excess weight, high blood pressure and depressive symptoms [1] compared to past surveys from 2006, 2012 and 2016 [14–16]. This article designed the Multidimensional Index in Health (MHI) which, using fuzzy sets, allows for the detection of the states with the greatest or least loss of health, via georeferencing, thus allowing for the dimensions of the public health problems to be observed from a global perspective. The vulnerability of some groups, such as people who consume alcoholic beverages and tobacco, is evident in this study as they generally scored 3 points above those individuals who do not smoke or ingest alcoholic beverages. Age is also one of the biggest factors with those in the 20–30 age group with an MHI of 17.13 while the group over 50 has an MHI of 31.3.

While obesity has been analyzed from many different perspectives, this study is unique in its use of a Multidimensional Health Index, which combines four study factors along with geo-referencing data at the national level regarding the status of the population. It is important to remember that excess weight, diabetes, depressive symptoms and high blood pressure, among others not analyzed in this study (e.g. stress, physical inactivity, inadequate diet, high cholesterol, lipids), are considered cardiovascular risk factors [17], and that these risk factors have claimed lives. The World Health Organization (WHO) [18] estimated in 2016 that at least 1.6 million people died from diabetes related complications worldwide, and that between 2000 and 2016, the mortality rate has increased by 5% in these patients.

Some aspects of this study are very interesting, among them that Jalisco, Querétaro and Tlaxcala are the states with the lowest MHI, unlike Sonora and Veracruz. On the other hand, it should be noted that higher levels of education tended towards lower MHI scores. Results relating to income level showed that excess weight and depressive symptoms were more prevalent among higher income individuals, unlike high blood pressure and diabetes, which decrease in this group. This information can be contrasted to existing literature separately considering that the MHI is a new index being proposed in this study.

Regarding the other factors being analyzed, starting with high blood pressure, a decreasing prevalence can be observed for this condition between ENSANUT 2012, ENSANUT 2016 and now ENSANUT 2018–19, which reported 27.2, 25.5 and 19.2% respectively. This is particularly intriguing, and merits further attention given that the other factors have been increasing in percentage [14, 15]. Likewise, the prevalence of high blood pressure was highest among the middle socioeconomic level in ENSANUT 2016, in contrast to ENSANUT 2018–19 which reports that the highest concentration of individuals with high blood pressure is now concentrated at the highest socioeconomic level [16].

Data regarding high blood pressure and educational level seems to be consistent since ENSANUT 2006, given that individuals with no education, or those with primary and secondary school, have a higher prevalence of hypertension, compared to those with a higher degree of education [14, 19].

Regarding the prevalence of hypertension by state, ENSANUT 2012 reported the states with the highest prevalence as Mexico City, followed by Baja California Sur, Chiapas, Tabasco, Oaxaca, Nayarit, Aguascalientes, however, ENSANUT 2018–19, just 6 years later, reports a significant change in this panorama with the highest prevalence being reported in the sates of Sonora, Veracruz, Campeche and Quintana Roo [15, 19].

Depressive symptoms, on the other hand, have increased compared to ENSANUT 2012, from 9.2 to 11.9%, representing an almost 3 % increase in the most recent ENSANUT 2018–19 survey. When this data is analyzed by sex, we find that the prevalence among women is 14.6% while the prevalence among men is 6.7%, demonstrating an increase with respect to that reported by Rafful in 2012, where the prevalence was determined to be 10.4% in women and 5.4% in men [20, 21] . This increase has been particularly significant in women, who are reportedly twice as likely than men to suffer from this condition, with those between the ages of 45 and 54 representing the most vulnerable group [20, 22]. Furthermore, if this data is correlated with diabetes mellitus, it has been shown that depression increases two to three-fold, tough many patients potentially remain undiagnosed [22–24].

On the other hand, evidence suggests that depression appears to be the result of a combination of socioeconomic and health factors, not exclusive to diabetes. Nevertheless, studies agree that women tend to have a higher prevalence of depressive symptoms than men, and that chronic degenerative diseases may play a role in increasing depressive symptoms and weight gain [25, 26].

This shows that depressive symptoms must be taken into account, especially in patients who have any of the four factors that were studied in this article, since these symptoms have been shown to affect glycemic control and adherence to medications among patients with diabetes [27].

In relation to sex, the MHI is more affected in women than in men, and this is also evident when each of the four study factors is analyzed by sex. If we take obesity or excess weight into account, women outnumber men in this study, an observation which is consistent with other articles, where obesity is reported to be higher among women, likewise, in surveys including ENSANUT 2006 [4].

On the other hand, reports from 2006 indicated that excess weight (i.e. overweight or obesity) exceeded 70%, while the ENSANUT 2018–19 survey indicates that the sum of overweight and obese (grades 1, 2, or 3) individuals now exceeds 80%, both in men and women, representing an increase of 10% according to the BMI of those surveyed [1, 3, 19]. In addition, as mentioned above, obesity is more prevalent among women than among men with 71.9 and 66.7% respectively. This data agrees with results reported by Jaacks in 2019 where, globally, the prevalence of obesity was shown to be higher among the female sex than the male sex [28]. Regarding excess weight and academic level, higher academic levels were associated with increased weight, which agrees with the data reported by Jaacks, in 2019, who published a report on the stages of the global obesity epidemic where Mexico was placed in stage 2, meaning that obesity is more prevalent among individuals of the higher socioeconomic levels, that obesity is found between 20 and 40% of the overall population, and that it is more prevalent among women than men [28]. This data also agrees with an article by Dinsa in 2012, where obesity was shown to be more prevalent among individuals of higher socioeconomic and educational levels in developing countries [29].

Regarding excess weight and its distribution by state, a study by Barrera Cruz [3] in 2013 described how adolescent and adult populations of the southern states such as Oaxaca and Guerrero had a lower prevalence for being overweight, compared to the northern states [3]. Currently, excess weight is more prevalent throughout the norther part of the country than in the southern part, with the exception of Campeche which has a higher prevalence [1]. It is evidenced that excess weight, especially obesity, is a factor that triggers chronic degenerative diseases, such as: metabolic syndrome, diabetes, high blood pressure, so it is a very important factor to take into account when measuring the state of health [22, 30], ^particularly^ considering the current magnitude of this condition.

Diabetes, the final factor, is one of the top 10 causes of death among adults [31]. The prevalence of this pathology has increased among the Mexican population in relation to the previous ENSANUT surveys of 2006, 2012, 2016 and now 2018–19. From 2006 to 2012, it has increased by 31.4%, and from 2012 to 2016, it only increased by 2.2%, while in the current ENSANUT 2018–19 survey, it affects 11% of the population, compared to 9.4% in 2016, representing an increase of 1.6%. It is important to consider that most of the population with diabetes or high blood pressure are also overweight or obese, a figure which is consistent with the previous ENSANUT in 2016 [32].

In 2019, the International Diabetes Federation (IDF) estimated the global projection for diabetes by 2030 and 2045. With a representation of 138 countries, the prevalence in 2019 was estimated to be 9.3%, with an expectation that this would increase to 10.2% by 2030 and 10.9% by 2045 [31]. In Mexico, through the ENSANUT 2018–19 survey, this projection is very visible, with a current prevalence of 11%, which is an alarming figure that is well above the global average. In terms of sex, the statistics in Mexico 2018–19 agree with those reported by Balakumar in 2016, where women have a higher prevalence than men [17]. However, although there is a higher prevalence of diabetes in women, there is evidence that mortality in men is higher than in women, particularly among 20 to 44 years olds [33].

In this sense, it is pertinent to mention that the results and discussion previously raised are widely subject to the type of data with which we work, that is, as we already explained in the methodological section, the main limitation lies in the adaptation of pre-collected data, which opens the opportunity for the development, validation and application of a new instrument which allows for the integration of concepts and / or elements with increased precision in the analysis of certain health factors.

## Conclusions

The relevance of the distinction, measurement and location of the factors that promote the loss of health of the population in Mexico is valuable, since it allows the distinction of key elements in the design of institutional and / or governmental strategies for improving the health of the general population.

The four health factors analyzed in this study are determinantal to the health of the inhabitants of Mexico, which is something that evidently follows international projections. It is important that these factors which have a negative impact on population health, such as excess weight, high blood pressure, diabetes and depressive symptoms, be studied using methods such as the construction of multidimensional indices, tools and / or other techniques such as the application of fuzzy sets.

The most current data on these four factors provided by ENSANUT 2018–19 show a growing and worrying prevalence, with the visibility of social groups with greater vulnerability and location in specific regions of Mexico, as well as gender-specific issues.

## Data Availability

The datasets generated and/or analyzed during the current study are available in the [INEGI] repository, [https://www.inegi.org.mx/programas/ensanut/2018/#Documentacion].

## Abbreviations

ENSANUT: National health and nutrition survey (ENSANUT 2018–19 - *for its acronym in Spanish*) in Mexico
WHO: World health organization
BMI: Body mass index
MHI: Multidimensional health index
